# The Privilege of Suffering

**Published:** 1886-11-13

**Authors:** 


*(To be continued.)*

### Words of Consolation.

[Communications for this column should be addressed, "Chaplain," care of the Editor of The Hospital.]

### VII.—The Privilege of Suffering.

Numbers of very good Christians readily admit what has been said about the privilege of suffering in the cases of prominent martyrs; but they do *not* quite see the same intention of God in the permission of sickness, or, say, in some of the common trials of home and social life. The result is a tendency to complain of anything like undeserved suffering. Suffering does not come under this head until it *is* undeserved. So long as it is deserved, or so long as it is purely of a disciplinary character, we can hardly call it a privilege. But, directly, we begin to wonder why this poor creature is given an apparently uneven share of suffering, or why that one is permitted to linger on, when in our opinion death would be the greatest mercy. Ah! then comes the need of some analysis of the great mystery of pain; then must we try and believe that even the prolongation of agony in certain cases has something to do with the martyr's privilege. We must therefore be slow to think that death could be any greater mercy if hastened so rapidly as we would have it.

"Meek souls those are who little deem
Their daily life an Angel's theme;
Nor that the Cross they view so calm
Shall prove in Heaven a martyr's palm."

Such a thought may also help to correct the very prevalent idea that when active work ceases the chief part of the Christian's warfare is accomplished. More often it is otherwise. More often those whom we consider shelved, or next to useless, are far more conspicuous in the sight of angels, are doing more to beat down Satan, and so to glorify God, than we ourselves in the midst of our, so called, labours. One of our bishops has well said: "It almost seems as if the members of Christ's body are to do yet more through their sufferings than through all their energizing, as if through the wounds of the members, as well as of the Head, life is to flow out."

Let any sufferer who may discern in the smallest degree the privilege we have discussed, think it possible, if the Father's voice were heard, that He might be saying, "Behold, I have created this My child, redeemed him with the precious Blood of My own dear Son, sanctified him with My Holy Spirit. Yea, I have chastened Him again and again; but I have not given him over unto death, because I want him yet to be My witness upon earth. The white robes of righteousness are his, if he will only keep them. But I want him yet to win the palm-branches in the joy of enduring for My cause in the world." Surely, to consent to such a high calling as this is one of the noblest acts of which the will of man can become capable. This is the only solution of joy and tribulation being so closely allied as they are in Holy Scripture, and in lives of the Saints. It is hard indeed "to count them happy which endure," unless we can grasp the grand idea of their being *selected* to vindicate God's honour in the face of the universe.

